# Exploration of the Diversity of Vicine and Convicine Derivatives in Faba Bean (*Vicia faba* L.) Cultivars: Insights from LC-MS/MS Spectra

**DOI:** 10.3390/molecules29051065

**Published:** 2024-02-29

**Authors:** Kjell Sergeant, Simon Goertz, Salma Halime, Hanna Tietgen, Hanna Heidt, Martina Minestrini, Cédric Jacquard, Stephanie Zimmer, Jenny Renaut

**Affiliations:** 1 Biotechnologies and Environmental Analytics Platform (BEAP), Environmental Research and Innovation Department (ERIN), Luxembourg Institute of Science and Technology (LIST), 5, Rue Bommel, L-4940 Hautcharage, Luxembourg; salma.halime@list.lu (S.H.); martina.minestrini@list.lu (M.M.); jenny.renaut@list.lu (J.R.); 2 NPZ Innovation GmbH, Hohenlieth-Hof 1, 24363 Holtsee, Germany; s.goertz@npz-innovation.de (S.G.); h.tietgen@npz-innovation.de (H.T.); 3 Université de Reims Champagne-Ardenne, INRAE, RIBP USC 1488, 51100 Reims, France; cedric.jacquard@univ-reims.fr; 4 Institut fir Biologësch Landwirtschaft an Agrarkultur Luxemburg a.s.b.l (IBLA), 1 Wantergaass, L-7664 Medernach, Luxembourg; heidt@ibla.lu (H.H.); zimmer@ibla.lu (S.Z.); 5 Louvain Institute of Biomolecular Science and Technology (LIBST), UCLouvain, Croix du 11 Sud 4-5/L7.07.03, B-1348 Louvain-la-Neuve, Belgium

**Keywords:** *Fabaceae*, alkaloids, convicine, vicine, fragment ion screening, dicarboxylic fatty acid, linkers

## Abstract

While numerous *Fabaceae* seeds are a good nutritional source of high-quality protein, the use of some species is hampered by toxic effects caused by exposure to metabolites that accumulate in the seeds. One such species is the faba or broad bean (*Vicia faba* L.), which accumulates vicine and convicine. These two glycoalkaloids cause favism, the breakdown of red blood cells in persons with a glucose-6-phosphate dehydrogenase deficiency. Because this is the most common enzyme deficiency worldwide, faba bean breeding efforts have focused on developing cultivars with low levels of these alkaloids. Consequently, quantification methods have been developed; however, they quantify vicine and convicine only and not the derivatives of these compounds that potentially generate the same bio-active molecules. Based on the recognition of previously unknown (con)vicine-containing compounds, we screened the fragmentation spectra of LC-MS/MS data from five faba bean cultivars using the characteristic fragments generated by (con)vicine. This resulted in the recognition of more than a hundred derivatives, of which 89 were tentatively identified. (Con)vicine was mainly derivatized through the addition of sugars, hydroxycinnamic acids, and dicarboxylic acids, with a group of compounds composed of two (con)vicine residues linked by dicarboxyl fatty acids. In general, the abundance profiles of the different derivatives in the five cultivars mimicked that of vicine and convicine, but some showed a derivative-specific profile. The description of the (con)vicine diversity will impact the interpretation of future studies on the biosynthesis of (con)vicine, and the content in potentially bio-active alkaloids in faba beans may be higher than that represented by the quantification of vicine and convicine alone.

## 1. Introduction

The *Fabaceae* or *Leguminosae* family, the third largest family of flowering plants, contains numerous species whose protein-rich seeds are of interest as food and feed or as a source of compounds/extracts for medical use [1,2]. The application range of legumes will furthermore likely increase due to the continued exploration of poorly used species [3] and the discovery of new/better activities in species that are already valorized. In human nutrition, either the entire pods or the seeds are consumed, with the latter making up the bulk of the consumption. Seeds from species such as soybean, peas, peanuts, lentils, and chickpeas are consumed worldwide and are an indispensable part of many local cuisines, providing protein, dietary fibers, and micronutrients. Furthermore, the capacity to enter into symbiosis with nitrogen-fixing bacteria makes the legumes low-input crops [4] that, depending on the species, also provide specialty phytochemicals such as isoflavones [5]. Globally, there is a growing interest in the cultivation of grain legumes, stemming not only from the demand for protein-rich food in vegan/vegetarian diets but also from supra-national efforts to promote low-input protein production such as the EU Protein Strategy (https://www.europarl.europa.eu/RegData/etudes/BRIE/2023/751426 (accessed on 23 September 2023)).

While legumes are thus attractive for cultivation and human consumption, some species also contain compounds with an “anti-nutritional” effect [6,7]. The protein-rich seeds are attractive for herbivores; therefore, legume seeds are stocked with a plethora of chemicals and proteins that inhibit digestibility to limit this biotic stress. These include saponins, the glycosides of steroids or triterpenes, which are studied for their antifungal activities [8]. Contrary to the saponins produced in numerous *Fabaceae*, some species contain specific metabolites that are toxic when consumed. Excessive or prolonged consumption of seeds from the grass pea (*Lathyrus sativus* L.) can cause neurolathyrism, a disease characterized by paralysis of the lower body and degenerative changes to the spinal cord. Neurolathyrism is caused by the neurotoxin l-β-*N*-oxalyl-α,β-diaminopropionic acid (β-ODAP) present in Lathyrus seeds [9], although the toxicology has most likely been oversimplified [10]. Another legume with seeds containing potentially toxic compounds is *Vicia faba* L., known as faba beans, fava beans, or broad beans.

Faba beans contain the fungicidal glycoalkaloids vicine and convicine [11], distributed over all plant parts [12] but with the highest concentrations in seeds, reaching 5 and 2 mg/g dry weight. In planta, vicine and convicine are part of the chemical defense against biotic stress, showing, for instance, an antifungal activity [11]. The hydrolysis of the glucose unit results in the release of the aminopyrimidine aglycones divicine and isouramil. After ingesting faba beans, individuals with a low-activity variant of glucose 6-phosphate dehydrogenase in erythrocytes suffer from favism, a life-threatening decrease in viable red blood cells [13,14]. The use of faba beans as animal feed is likewise limited, as this is known to impact productivity negatively, for instance, in poultry farming [15]. Contrary to this, the abundance of vicine and convicine seems to have a negligible impact on weaner pig performance, which is influenced by other antinutritional factors present in the faba beans [16]. The presence of these compounds thus limits the cultivation and use of this otherwise excellent source of protein, carbohydrates, fiber, and interesting phytochemicals, which, furthermore, has highly favorable agricultural and ecological properties [17,18]. Due to the limitations imposed by these compounds, plant breeders use vicine/convicine content as a selection target [19,20], with a low vicine–convicine faba bean line that has been available for 40 years. As an alternative, enzymatic methods have been developed to eliminate vicine and convicine from food matrices that contain faba beans [13,21].

Numerous analytical approaches have been developed for the analysis of glycoalkaloids [22] and specifically for the quantification of (con)vicine. Initially, these methods were based on spectrophotometry [23] and HPLC with UV detection [24]. As for the analysis of natural products in general [25], most current methods for (con)vicine analysis use mass spectrometry hyphenated to HPLC [26]. The HPLC operated is mostly reversed-phase [13], while the hydrophilic nature of vicine and convicine also allows the use of normal-phase separations, for instance, with HILIC [26,27]. However, the highest throughput was obtained using flow injection MS/MS, resulting in a 60 s analysis time [28].

(Con)vicine derivatives were described for the first time in 2021 [29] but had already been annotated as derivatives without further identification in 2019 [30]. Kowalczyk et al. (2021) describe 11 compounds, of which 7 were identified as (con)vicine derivatives based on the characteristic signal of divicine and isouramil in positive mode. During the metabolite profiling of faba bean seedlings with UPLC-MS/MS, we recognized a number of compounds that present the characteristic fragments of vicine and convicine [28]. We realized that the diversity of (con)vicine-based compounds is higher than that described in the literature and that this may have an impact on studies on their biosynthesis; therefore, the data were systematically screened for MS/MS spectra with *m*/*z* 141.018 and 141.040 base peaks. This resulted in more than 130 hits, of which the majority were identified based on observed mass shifts; however, the exact connectivity between the different groups could not be established. From the dataset, it is observed that the relative MS signal intensity over the five varieties of most, but not all, derivatives follows that of the mother compounds. We present here the identification of a collection of derivatives of these highly studied compounds, including compounds composed of two (con)vicine residues connected with different dicarboxylic acids.

## 2. Results

While the negative ion of isouramil is expected to generate an *m*/*z* value of 142.026 ([M − H]^−^), the *m*/*z* observed experimentally is 141.018. This has been reported previously for the negative mode LC-MS/MS identification of these compounds [30]. Purves et al. developed a Multiple Reaction Monitoring (MRM)-based method for the quantification of vicine/convicine and used the transitions 303 to 141 for vicine and 304 to 141 for convicine [28]. The calculated mass for C_4_H_3_N_3_O_3_, the [M − 2H]^−^ ion of isouramil, is 141.017, which corresponds to the *m*/*z* of 141.018 that we observed. For vicine, the observed base peak fragment ion is 141.042, corresponding to C_4_H_5_N_4_O_2_ or the [M − H]^−^ ion of divicine.

**Figure 1:**
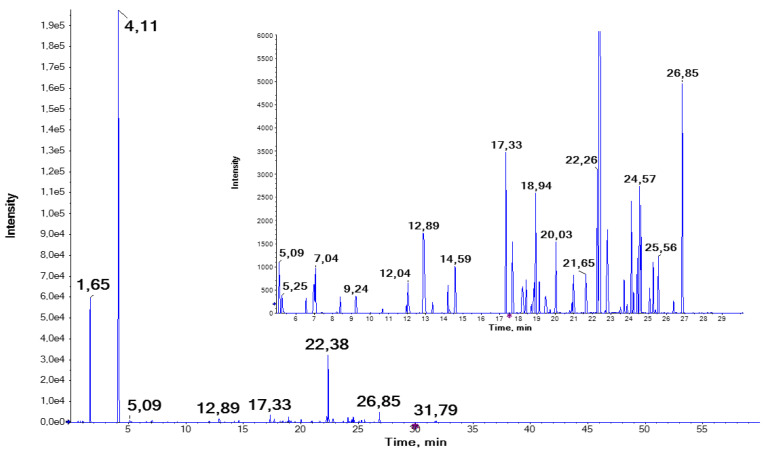
Extracted fragment ion chromatogram of the fragment at 141.03 ± 0.015, thus covering the base peak fragment of vicine and convicine. The inset shows the RT range from 5 to 30 min; the peak at 22.38 is cut to illustrate the number of MS/MS spectra containing the fragment at 141.03 ± 0.015. Output generated during the data analysis with Progenesis QI v2.3.

After the identification of vicine and convicine (compounds **1** and **2**, Table 1), hereafter called the mother compounds, based on these characteristic fragment ions, and a comparison to published MS/MS spectra, it was observed that other compounds showed the same fragments. To extract potential (con)vicine derivatives, the data were filtered for the presence of the fragment at *m*/*z* 141.03 ± 0.015 (covering both vicine and convicine) using the PeakView fragment filter tool (version 2.2.0.11391, AB Sciex). The extracted fragment ion chromatogram generated is shown in Figure 1. A manual inspection of the MS/MS spectra, at which the fragment at 141.03 was observed, resulted in the identification of 163 features containing either vicine or convicine. The convicine-based compound *m*/*z* 720.322 at RT 27.08 is the derivative with the highest retention time. Compounds based on (con)vicine span the retention time range from 0.5 to 27.08 min and an *m*/*z* range from 303 to 1156.

**Figure 2:**
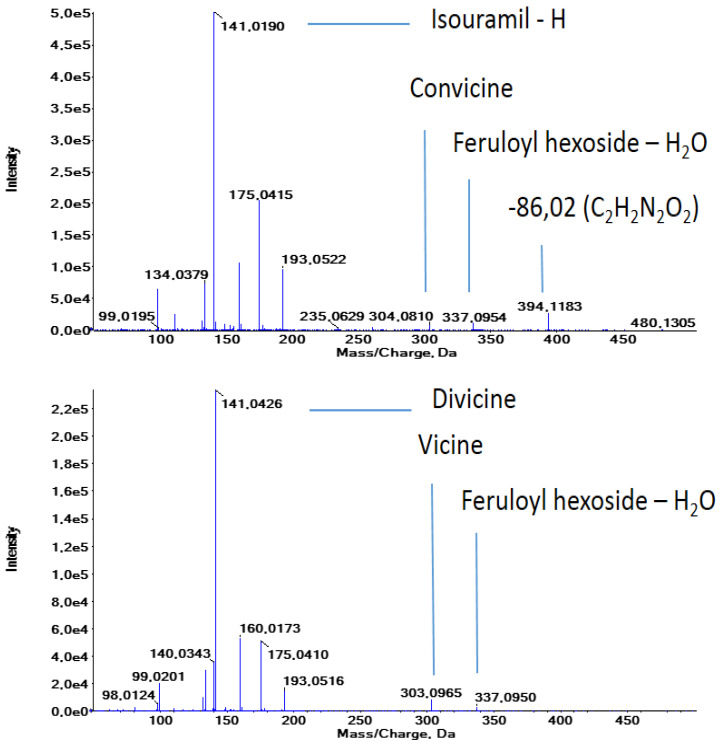
The MS/MS spectra of feruloyl convicine and feruloyl vicine indicated are the characteristic fragment ions for the two gluco-aminopyrimidines and feruloyl glucoside showing the attachment of ferulic acid to the glucose. Other major fragments at *m*/*z* 193, 175, 160, and 134 correspond to ferulic acid fragments.

At a retention time of 19.58 min, the *m*/*z* 141.03 fragment is observed in the MS/MS spectra of the compounds at *m*/*z* 480.124 (compound **38**, Table 1) and 961.256 (dimer of 480.124). The exact *m*/*z* of 141.017 and 304.077 allows us to identify convicine, while the remainder of the highest fragment peaks (193.05, 175.04, 160.015, 134.036, and 132.02, Figure 2) agree with the MS/MS spectra of a ferulic acid standard and published data on ferulic acid conjugates [31]. Compound **38** is thus feruloyl convicine (calculated *m*/*z* 480.126; Figure 2, upper spectrum), one of the convicine derivatives previously identified [29]. The vicine homologue (compound **39**; *m*/*z* 479.140; calculated *m*/*z* 479.142) was subsequently identified at a retention time of 19.95 min (Figure 2, lower spectrum). These identifications were confirmed in positive mode.

There is no fragment with an *m*/*z* corresponding to the ferulic acid attached to the aminopyrimidine moiety. However, in both MS/MS spectra in Figure 2, *m*/*z* 337.091, corresponding to [feruloyl hexoside − H_2_O], is observed. This shows that the ferulic acid is attached to the glucose of (con)vicine. The fact that derivatization occurs via the sugar moiety is also confirmed by the observation of the neutral loss of 124.039 amu, corresponding to [divicine − H_2_O], from most vicine-based compounds. For instance, for compound **50** (Table 1), identified as vicine-diferuloyl hexoside, an in-source fragment with *m*/*z* 693.197 (Δm = 1.2 ppm from the calculated *m*/*z* of the compound **50** with the in-source loss of divicine) is observed. This confirms that no derivatization takes place on the aminopyrimidine moiety of vicine. The analogy between vicine and convicine and the fact that the same derivatives are found for both mother compounds, allows us to hypothesize that convicine is also derivatized on the glucose moiety.

The gross composition of more than half of the recognized LC-MS/MS features containing (con)vicine could be identified (that is, the groups attached to the mother compounds could be determined), while limited data were obtained on how the groups are linked. Therefore, the identifications reported are tentative, and confirmation by using either standards or NMR is needed. The elimination of those features corresponding to neutral losses and dimer formation resulted in the identification of 89 vicine-/convicine-based compounds, represented in Table 1. In Supplemental Table S1, 42 compounds are represented that contain either vicine or convicine but for which no further identification of the derivatives was possible.

The compounds in Table 1 can be separated into three different groups. The first group (compounds **1** to **25**) comprises derivatives with small molecules, with a maximum of six carbons and at least one carboxyl function. The second group of (con)vicine-based compounds are derivatives with phenylpropanoids or guaiacylglycerol (compounds **26** to **52**). Most striking in this group are the derivatives containing multiple ferulic acid and hexose residues (compounds **45** to **52**). The MS/MS spectra of the compounds resemble the spectra shown in Figure 2, with little to no MS/MS fragments observed above *m*/*z* 479/480 (vicine/convicine ferulic acid). This lack of ions precludes the discernment of connectivity between the different groups. Only in the MS/MS spectra of *m*/*z* 1155 (compound **52**) is some further structural indication observed. Low-intensity fragment ions are observed at 479.146 (vicine ferulic acid) + 162 and 479.146 + 176, which may indicate the formation of a branched rather than a linear ferulic acid–glucose chain. No derivatives were found in which a combination of ferulic acid and hexoses connects two aminopyrimidines.

The third group contains (con)vicine derivatized with groups with more than six carbons, or with such group of more than six carbons linking two (con)vicine residues. The first identification in this group were compounds wherein the derivatizing group is attached to a single (con)vicine, and the formula reported corresponds to the “(con)vicine + the formula of the derivatizing group − H_2_O”. To illustrate that some of the compounds are formed by a bifunctional linker and two (con)vicine residues, this formula is maintained, and identification is thus reported as “(con)vicine + the formula of the derivatizing group − H_2_O (con)vicine”, although the linker obviously loses an additional H_2_O to form the link with the second (con)vicine.

**Figure 3:**
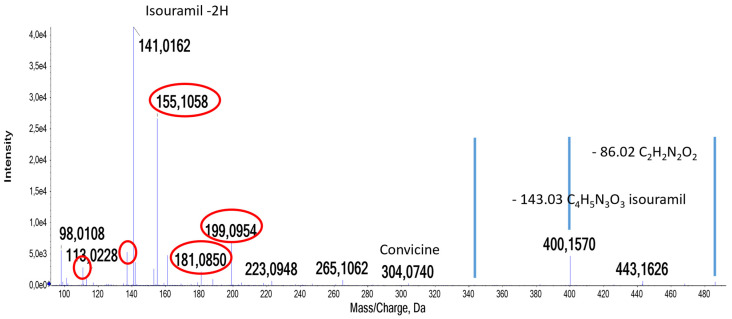
MS/MS spectrum of compound **84** (*m*/*z* 486.172 RT 22.49), tentatively identified as convicine + C_10_H_14_O_3_; characteristic losses for convicine are indicated: the loss of 86.03 (C_2_H_2_N_2_O_2_) from isouramil, the loss of isouramil (−143.03 C_4_H_5_N_3_O_3_), convicine at *m*/*z* 304.074, and isouramil − 2H at *m*/*z* 141.016. The highest intensity *m*/*z* values that were used in the MZCloud library search and matched to decenedioic acid are encircled in red.

This group of compounds is illustrated by the identification of vicine + C_10_H_14_O_3_, convicine + C_10_H_14_O_3_, and both alkaloids linked with this derivative. In Figure 3, the MS/MS spectrum of compound **84** is shown. The base peak is formed by the fragment at *m*/*z* 141.018, and a minor peak is observed at 304.074, indicating that this is a convicine-based compound. The mass difference between the experimental mass at *m*/*z* 486.172 and the calculated mass for convicine is 182.094 amu; this corresponds to the formula C_10_H_14_O_3_ (mass error of 1.6 ppm). When the water lost during the formation of the bond with vicine is added, this becomes C_10_H_16_O_4_, a group observed in the MS/MS spectrum at *m*/*z* 199.098 (mass error of 3.9 ppm). From the fragment at 199, there is a loss of 43.994 amu, corresponding to CO_2_. A similar MS/MS is reported for decenedioic acid [32], with a matching chemical formula. The fragment peaks shared between compound **84** and compound **85**, and not present in the MS/MS of vicine or convicine, were used in a library search with MZCloud™. The highest-scoring compound from this library search is 2-decenedioic acid, with a formula that matches the observed added mass. A further comparison of the MS/MS spectra of these compounds in both ion modes with available predicted spectra (for instance HMDB0000603) confirms that fragmentation spectra from *m*/*z* 486.172 are indeed as expected for convicine linked to decenedioic acid on the glucose moiety.

**Figure 4:**
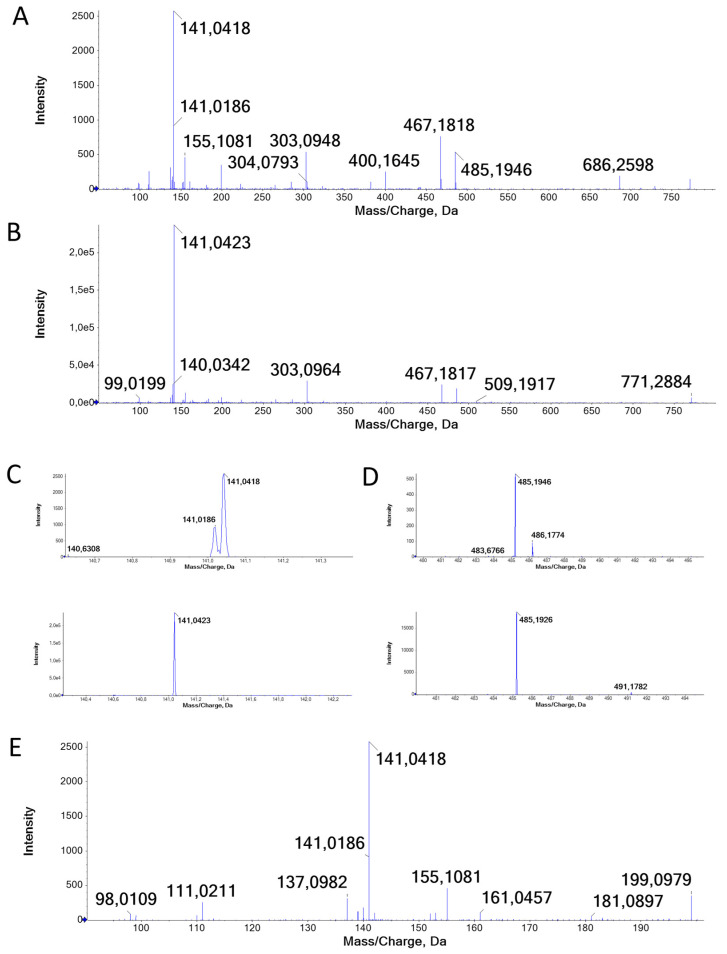
Spectra resulting in the identification of the MS features (21.94, 772.26) and (22.17, 771.28) as a convicine and a vicine linked by decenedioic acid and two vicines linked by decenedioic acid, respectively. Spectra (**A**,**B**) show the total fragmentation spectrum, while spectra (**C**–**E**) show details of these spectra.

The derivatizing molecule resulting in the compound shown in Figure 3 is confirmed to be a dicarboxylic acid through the identification of compounds wherein two (con)vicine residues are linked. At RT 21.94 and 22.17, *m*/*z* 772.265 and 771.279 are fragmented (compounds **76** and **80**, respectively, Figure 4A,B). In Figure 4A, the peak at *m*/*z* 686.260, loss of 86.03 amu, indicates that convicine is present, while the MS/MS base peak at 141.04 indicates vicine, as does *m*/*z* 303.09, the negative ion of vicine. These two latter fragments are also observed in Figure 4B, while no peak due to the loss of 86.03 is observed.

Details of these spectra confirm that compound **76** indeed contains both vicine and convicine. A detail of spectra Figure 4A shows that both 141.042 and 141.019, characteristic for vicine and convicine, respectively, are observed (Figure 4C, upper spectrum), while in the lower spectrum, a detail of Figure 4B, only the fragment indicating the presence of vicine is observed. Similar to this is the loss of both vicine and convicine, resulting in fragments at *m*/*z* 486.177 and 485.195 (Figure 4D upper spectrum), observed in the detail of Figure 4A. In the detail of spectrum 4B, only the loss of vicine to *m*/*z* 485.193 is observed (Figure 4D lower spectrum), confirming that compound **80** contains only vicine. Figure 4E shows the lower mass range of Figure 4A, illustrating that the same fragments that were used for the tentative identification of decenedioic acid as the derivatizing molecule of the compound **84** (Figure 3) are present. Taking all this into account, compound **86** is tentatively identified as a vicine linked to a convicine by decenedioic acid. Compound **80** is then two molecules of vicine linked by decenedioic acid.

In a similar way, the other compounds of the third group of (con)vicine derivatives in Table 1, dicarboxylic fatty acids bound to a vicine or convicine or forming a linker between different combinations of these, were identified. To make it clear that these different forms exist, the molecular formula of the dicarboxylic acid with the loss of a single water molecule was used as the annotation.

**Figure 5:**
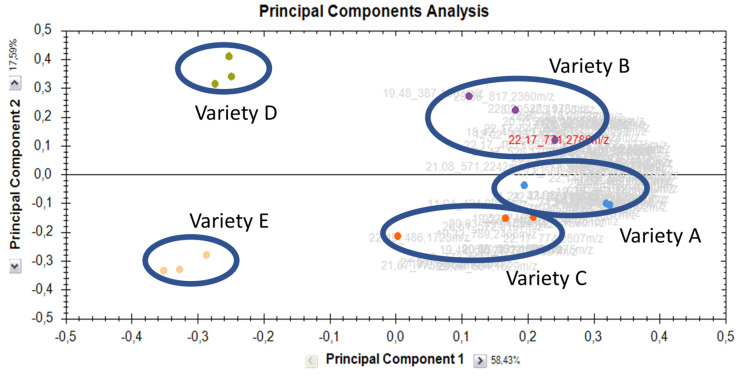
PCA plot calculated based on MS signal intensities of the compounds reported in Table 1; Output generated during the data analysis with Progenesis QI v2.3. The capitals A to E represent the five varieties used in this study for which details are provided in Table 2.

The 89 compounds represented in Table 1 were used for quantitative analysis. The principal component analysis with these compounds resulted in the separation of varieties D and E from the others on the first principal component, accounting for 58% of the variance (Figure 5). Varieties D and E are separated on the second principal component, accounting for 18% of the variance. Varieties A to C show a higher variance among the replicates and are less well separated on the PCA plot (Figure 5). Most of the compounds follow the profile of the mother compounds, that is, high in varieties A, B, and C and low varieties in D and E. For further quantitative assessment, more analyses are needed, as well as the use of standards for absolute quantification. However, the high MS signal intensities recorded for some of the derivatives reported here indicate that they are present in higher quantities than trace amounts.

## 3. Discussion

In total, 163 LC-MS features containing one or more convicine or vicine groups were recognized. After eliminating dimers and features due to neutral losses, 131 LC-MS features remained. Of these, the gross structure could be established for 89 compounds, indicated in Table 1 with quantitative data, with the main fragment ions indicated in Supplemental Table S2. Based on the characteristic fragments, the presence of vicine and/or convicine could be established for the 42 remaining compounds (Supplemental Table S1). Although details of the connectivity between the different groups in these compounds could not be established, for all compounds for which this could be observed, derivatizing groups were bound on the sugar moiety of (con)vicine. The relative quantification of the compounds in the different varieties was performed based on the MS signal intensity. Although this is only a proxy for the abundance of a compound, it allows a relative cross-variety comparison of the abundance of a compound. The MS signal intensity data presented in Supplemental Table S2 can therefore only be interpreted as such.

The first group of derivatives in Table 1 (compounds **1** to **25**) are derivatives found on numerous other groups of biomolecules. They include malonic, (iso)valeric, and benzoic acid, which are relatively common on biomolecules, including on glycosylated flavonoids isolated from *Fabaceae* [33]; some of these (con)vicine derivatives were previously tentatively identified in faba bean seeds [29,30]. The identification of these compounds is supported by well-described observations such as the loss of CO_2_ from malonyl derivatives in negative mode [34] or known masses for derivatives (among others, those described in maize [35]).

Compounds **26** to **52** are derivatives with hydroxycinnamic acids. The highest MS signal intensity was observed for derivatives with ferulic acid (Figure 2). As for the first group of derivatives, derivatives of biomolecules with hydroxycinnamic acids are commonly found in plants. Relatively complex combinations of hydroxycinnamic acids and sugars have been identified on faba flavonoids [36]. Flavonoids carrying derivatives as complex as compounds **45** to **52** were identified in kale [37], and Arabidopsis anthocyanins are known to contain large chains composed of hydroxycinnamic acids and sugars. In the latter case, the addition of hydroxycinnamic acid groups influences the absorption spectrum of the anthocyanins, modulating their protective activity [38].

The third group of compounds (compounds **53** to **89**) are tentatively identified as (con)vicine with dicarboxylic acids or two molecules of (con)vicine linked by dicarboxylic acids. While derivatives with short-chain dicarboxylic acids such as malonic, succinic, and (hydroxy)adipic acids are known for a range of biomolecules and are found among the (con)vicine derivatives in Table 1 (compounds **5** to **9**), derivatives of biomolecules with longer dicarboxylic acids are rare. One group of such compounds is dicarboxylic acids conjugated to amino acids and amines, such as carnitine [39]. In humans, these acylcarnitines are involved in the transport of fatty acids to the mitochondria, and a transport, stabilizing, and/or protective function can be hypothesized for these (con)vicine derivatives.

The PCA analysis, shown in Figure 5, illustrates that quantifying these 89 compounds in shoots of 14-day-old seedlings allows us to differentiate varieties classified as low-VC from high-VC varieties (Table 2). As is known and illustrated in our dataset, this can also be performed using standardized methods for the absolute quantification of vicine and convicine [26,28]. A correlation analysis of the 89 derivatives in Table 1 resulted in 5 clusters. In cluster 1, 20 derivatives are found that are high in A and B and low in C–E. Vicine (compound **1**) is in this cluster, and the 19 other compounds in this cluster all contain vicine, with only compound **55** being convicine linked to vicine. The abundance of compounds in this cluster is thus driven by the abundance of vicine. Contrary to this, cluster 2 contains compounds high in varieties A, C, and to a lesser degree, E. The compounds in this cluster are convicine linked to convicine (compounds **56**, **70**, and **73**) or vicine (compound **79**). Surprisingly, these are higher in the three varieties with the lowest convicine/vicine ratio. Cluster 4, with 6 compounds that have a minimum MS signal intensity in shoots from variety B, includes both hydroxybenzoic acid and both dihydroxy-tetradecadienedioic acid derivatives (compounds **17**, **19**, **62**, and **66**). The remaining derivatives follow the description of the varieties (Table 2) and the abundance profile of the mother compounds (Supplemental Table S2), with the majority found in cluster 5. The general observation is thus that the MS signal intensity of derivatives follows that of the mother compounds with only eleven derivatives (clusters 2 and 4) that have a clearly different profile.

Based on the tentative identifications presented in Table 1, some general observations can be made. Convicine derivatives have a lower retention time than the corresponding vicine derivative. For most derivatives, the MS signal for convicine derivatives is higher than that of the vicine derivative. The absolute quantification of the mother compounds in different tissues shows that vicine is higher than convicine in the seeds and stems, a ratio that is reversed in the roots [40,41]. The higher MS signal intensity of convicine observed here may be explained by the better ionization of this convicine derivatives.

To validate the presence of these compounds, different samples were created, and their metabolomic datasets were searched with our in-house database containing the 89 compounds in Table 1. In the roots of the five varieties used here, the same (con)vicine derivative were recognized (Supplemental Figure S1A,B), albeit with different ratios. Finally, the identifications were checked against a dataset of faba bean seedlings grown in-house. Extracts were made from the seedlings of 18 varieties of faba beans involved in an annual variety trial of grain-Leguminosae, germinated on pleated paper. The extracts were pooled to obtain an overall view of the different (con)vicine derivatives present, and an analysis was carried out as described in the Section 4. The derivatives presented here were also identified in this dataset (Supplemental Figure S1C,D). Contrary to the samples from NPZ on which this study is based, the samples used for verification were grown and prepared at LIST, thus avoiding artefacts that may be caused during transport. The identification of these derivatives in completely independent samples shows that these derivatives are not artefacts and can be expected to be present in all extracts from faba bean seedlings. Finally, datasets from extracts of seedlings of other *Fabaceae* (*Pisum sativum*, *Glycine max*, *Lupinus* spp., *Cicer arietinum*, and *Medicago sativa*) were explored and, as expected, based on the absence of the mother compounds in these species, no identification of (con)vicine or their derivatives was obtained.

The origin of these derivatives is not known. The key enzyme in the biosynthesis of (con)vicine is only expressed in the seeds of faba plants [12]. However, in the Supplemental Data of this publication, the characteristic fragment ions for vicine (RT 1.065 *m*/*z* 305 and 143) are found in all tissues, although at a low MS signal intensity [12]. Other studies quantified vicine and convicine in germinating seeds, stems, and roots at different developmental stages. Vicine and convicine were found in most tested tissues including vegetative excluding vegetative roots and stems [41]. Given the dry weight of vegetative-stage plants compared to seeds, it is unlikely that the redistribution of (con)vicine from seeds is the only source of these compounds [41]. Older studies calculated the total amount of (con)vicine present in seeds and in vegetative tissues and discovered that not all (con)vicine found in vegetative tissues can originate from the amounts present in the seed, indicating de novo synthesis in *V. narbonensis* [40,42]. For *V. faba*, a first study indicated that the sum of vicine and convicine present in the seeds accounted for the amounts found in the roots, but would require the conversion of vicine to convicine [40]. This conclusion was later revoked by the same group, showing that both *Vicia* species must have the capacity to synthesize vicine and convicine in young plants [42]. With the addition of the derivates described here, the amount of divicine and isouramil in seedlings is much higher than represented by only vicine and convicine. The absolute quantification of the -(con)vicine derivatives described here in all tissues including seeds potentially resolves these contradictions.

So far, few (con)vicine derivatives have been identified in faba bean seeds. Valente et al. reported five derivatives and an unidentified compound that is likely also a vicine derivative [30]. Compound 33, reported by Valente et al., *m*/*z* 387 and its [2M − H]^−^ ion at *m*/*z* 775, is identified here as (iso)valeryl vicine (compound **24**, Table 1), also identified in [29]. Compound 59 in Valente et al., *m*/*z* 527, is identified here as vicine + C_12_H_16_O_4_ (compound **77**, Table 1). For two of the (con)vicine derivatives reported by Valente (compound 75 (*m*/*z* 553) and compound 37 (*m*/*z* 525)), no matching feature could be found in our data, as is also the case for the vicine pentoside reported by Kowalczyk [29]. While this may be due to the presence of seed-specific derivatives or the specific variety used in the different studies, it indicates that the overall diversity in (con)vicine derivatives is higher than described here. The analysis of more *Vicia* tissues from more varieties will likely result in the discovery of other derivatives.

Whether the (con)vicine derivatives reported in this study have an impact on the quality of food containing faba beans cannot currently be excluded and requires further study. First, although some (con)vicine derivatives have been identified in seeds, the total diversity of derivatives in seeds is not known. Furthermore, the anti-nutritional effects of vicine and convicine are caused by the free aminopyrimidines. Further research is needed to establish whether these derivatives contribute to the pool of bio-active divicine and isouramil in food or after digestion of faba beans containing food or feed.

In conclusion, in this paper, we described the diversity in compounds containing vicine and convicine, which is found reproducibly in the shoots and roots of *Vicia faba* seedlings. Three groups of compounds can be discerned, of which the third one, derivatives of (con)vicine with bifunctional molecules forming a linker between two (con)vicine residues, is a type of compound that has not been described previously. In general, for the five varieties analyzed, the MS signal intensity of the derivatives follows that of the mother compounds. To establish the impact of these derivatives on current knowledge of faba beans requires more research; the identifications need to be confirmed and refined. Secondly, detailed studies are needed to determine whether, and if so how, these (con)vicine compounds impact the amount of divicine and isouramil present in food containing faba beans. Because vicine and convicine relate to biotic stress [11,43,44], detailed in planta studies are needed to understand the in planta functions of the compounds reported here.

## 4. Materials and Methods

### 4.1. Plants and Sampling

In this trial, five different cultivars from the breeder Norddeutsche Pflanzenzucht KG (NPZ) (Holtsee, Germany) were analyzed. Four cultivars or varieties are officially registered in Germany: Taifun (2011), Trumpet (2017), Augusta (2018), and Allison (2019). The fifth variety, “E”, is a non-public breeding line. All varieties are spring types, except for Augusta, which is a winter type. The Taifun and “E” varieties are white-flowering (Zt) and thus tannin-free, and the other three varieties, Augusta, Trumpet, and Allison, are color-flowering (Bb) and tannin-rich. Based on the breeder-internal vicine threshold, the Taifun, Augusta, and Trumpet varieties are classified as high in vicine (HVC) and the Allison and “E” varieties as low in vicine (LVC).

From each cultivar, 30 seeds were randomly selected from a field-produced seed lot and seedlings were grown in the NPZ seed quality laboratory. The standard method for ISTA germination was utilized. Thirty seeds were sown in sand substrate in a plastic box and put in a climate chamber at 20 °C with 16 h under light and 8 h under dark conditions. The boxes were covered with black foil for seven days, before the foil was removed, and the seedlings were grown for another seven days, corresponding to the end of the sprout stage [41]. Three replicates of ten seedlings were sampled; sand was washed off the root system, and the plants were transferred to a closed plastic bag on ice packs. Upon sampling, the fresh seedlings in plastic bags were packaged and shipped per express delivery to LIST in Hautcharage, Luxembourg.

### 4.2. Metabolite Extraction

Upon arriving at LIST, the samples were lyophilized for 48 h using a Lyocube (Christ, Osterode am Harz, Germany). The seed parts still attached to the shoot were removed, and the lyophilized vegetative tissue was separated into shoots and roots. The respective organs of three individual plants were pooled to form one biological replicate. For each variety, three replicates were formed, the plants were ground in liquid nitrogen with a mortar and pestle, and the powder obtained was stored at −20 °C prior to extraction.

Approximately 100 mg of powder was weighted and 80% MeOH/20% MQ (Milli-Q water, Merck Millipore, Darmstadt, Germany) was added in a 1/12 ratio *w*/*v*; this was incubated at 22 °C with 1450 rpm agitation. After 4 h, the samples were centrifuged at 20,000× *g* for 30 min at 4 °C. The supernatant was recovered and dried under vacuum, after which the dried extract was stored at −20°C prior to analysis.

### 4.3. UPLC-MS/MS and Identification

The dried extracts were resolubilized in 5% MeOH/MQ in the same volume used for extraction and filtered through a 0.22μm PTFE syringe filter (Millex-LG, Merck KGaA, Darmstadt, Germany). The filtered extracts were analyzed using an Acquity UPLC I-class UPLC system equipped with a diode array detector (Waters, Milford, MA, USA) hyphenated to a TripleTOF 6600 (SCIEX, Framingham, MA, USA) mass spectrometer as previously described [45]. Five μL of the extracts was injected and separated on a reversed-phase column (Acquity UPLC BEH C18 column 2.1 × 100 mm, 1.7 μm particle size; Waters) with the column temperature set at 50 °C and a flow rate of 0.5 mL/min. The eluents were 0.1% (*v*/*v*) formic acid in water (A) and 0.1% (*v*/*v*) formic acid in acetonitrile (B) with the following gradient: 0 min, 1% B; 4 min, 1% B; 16 min, 5% B; 35 min, 40% B; 45 min, 100% B; 50 min, 100% B; 54 min, 1% B; 60 min, 1% B.

Electrospray ionization (ESI) was performed using the following parameters: source temperature 650 °C; ion spray voltage of 4.5 and −4.5 kV; curtain gas (nitrogen) of 30 psi; nebulizer gas (air) of 55 psi; and turbine gas (air) of 50 psi. The declustering potential was set up at 60 V and −60 V in positive and negative modes, respectively. The precursor charge state selection was set at 1. For information-dependent acquisition, MS scans (from *m*/*z* 100 to 2000) were acquired for 175 ms and the 10 most abundant *m*/*z* values were selected for MS/MS scans (from *m*/*z* 50 to 2000) of 200 ms. A sweeping collision energy of 15 V below and above 15 and −15 V, for the positive and negative modes, respectively, was applied to all precursor ions. After three occurrences, an *m*/*z* value was excluded for MS/MS for 2 s. Data for all replicates were acquired in negative mode, while positive mode analysis was run on one replicate of each couple variety × organ, and positive mode data were used to aid in the identification of the compounds.

For the identification and relative quantification, the raw data files were uploaded in Progenesis QI (v2.3, Nonlinear Dynamics, Newcastle upon Tyne, UK) in order to align all runs, normalize the data, and carry out a quantitative analysis based on the groups of samples (varieties). A separate work file was created for the roots and shoots. LC-MS features (retention time × *m*/*z* couples) without fragmentation spectrum were filtered out, as were peaks corresponding to known or suspected contaminants; the remaining features were used for identification. Initially, msp files downloaded from NIST, Massbank, and from our in-house database were used, and hits were manually validated. Compounds not found in these databases were identified through a literature search and MS/MS comparison with the following databases: LipidMaps (https://www.lipidmaps.org/); HMBD (https://hmdb.ca/spectra/ms/search) GNPS (https://gnps.ucsd.edu/ProteoSAFe/libraries.jsp); MZCloud™ (https://beta.mzcloud.org/); PubChem (https://pubchem.ncbi.nlm.nih.gov); and Metlin (https://metlin.scripps.edu).

For the semi-targeted identification reported here, a hypothetical identification was proposed after manual spectral interpretation; the structure was generated in Chemdraw (v: 19.0.0.22) and the theoretical MS/MS spectrum of this structure, generated by MetFrag (https://ipb-halle.github.io/MetFrag/) and CFM-ID (https://cfmid.wishartlab.com/) [46,47], were compared to the experimental spectrum. Accepted identifications were subsequently added to our in-house database for dereplication. All identifications reported here are at level 2 and are based on the *m*/*z* of the precursor and the comparison of the experimental MS/MS with experimental or predicted MS/MS spectra from the sources cited above [48].

## Figures and Tables

**Table 1:** Overview of the 89 (con)vicine derivatives identified in negative ion mode in the five faba bean varieties.

#	RT ^a^	*m*/*z* ^b^	Formula	FC ^c^	Max ^d^	Min ^d^	ID	ΔM ^e^	Derivative ^f^
mother compounds and short-chain acid (# C from 3 to 6) derivatives									
**1**	0.71	303.094	C_10_H_16_N_4_O_7_	7.5	B	E	VC	−0.8	
**2**	0.71	304.079	C_10_H_15_N_3_O_8_	5.7	B	E	CV	2.3	
**3**	1.10	466.131	C_16_H_25_N_3_O_13_	9.6	C	D	CV Hex	−1.9	
**4**	1.14	465.145	C_16_H_26_N_4_O_12_	7.6	C	D	VC Hex	−5.8	
**5**	1.49	390.078	C_13_H_17_N_3_O_11_	104.3	B	E	malonyl CV	−1.7	
**6**	1.69	389.093	C_13_H_18_N_4_O_10_	54.5	B	E	malonyl VC	−4.6	
**7**	3.64	448.120	C_16_H_23_N_3_O_12_	254.0	B	E	hydroxyadipoyl CV	−2.9	
**8**	4.19	447.136	C_16_H_24_N_4_O_11_	96.6	B	E	hydroxyadipoyl VC	−2.1	
**9**	4.62	448.118	C_16_H_23_N_3_O_12_	487.5	B	E	hydroxyadipoyl CV	−5.9	
**10**	5.64	409.097	C_16_H_18_N_4_O_9_	3.2	B	D	CV + C_6_H_3_NO	−7.9	nicotinic acid
**11**	6.19	404.130	C_15_H_23_N_3_O_10_	4.1	A	E	CV + C_5_H_8_O_2_	−3.8	hydroxy-valerate
**12**	6.42	360.104	C_13_H_19_N_3_O_9_	3.4	A	D	CV + C_3_H_4_O	−3.2	propionic acid
**13**	6.54	408.114	C_16_H_19_N_5_O_8_	5.4	B	E	VC + C_6_H_3_NO	−5.1	nicotinic acid
**14**	7.01	403.145	C_15_H_24_N_4_O_9_	4.0	A	E	VC + C_5_H_8_O_2_	−5.0	hydroxy-valerate
**15**	8.30	403.145	C_15_H_24_N_4_O_9_	4.9	B	E	VC + C_5_H_8_O_2_	−4.5	hydroxy-valerate
**16**	8.50	585.170	C_23_H_30_N_4_O_14_	9.6	A	E	VC + hydroxybenzoic acid Hex	1.6	
**17**	11.04	424.099	C_17_H_19_N_3_O_10_	4.3	A	B	CV + hydroxybenzoic acid	−2.4	
**18**	11.63	374.120	C_14_H_21_N_3_O_9_	3.8	C	E	butyryl CV	−0.5	
**19**	12.02	423.112	C_17_H_20_N_4_O_9_	2.5	A	E	VC + hydroxybenzoic acid	−8.1	
**20**	12.76	373.136	C_14_H_22_N_4_O_8_	4.0	C	E	butyryl VC	−1.1	
**21**	15.77	386.119	C_15_H_21_N_3_O_9_	3.9	A	D	CV + C_5_H_6_O	−4.2	pentenoic acid
**22**	17.44	385.134	C_15_H_22_N_4_O_8_	2.7	B	E	VC + C_5_H_6_O	−5.2	pentenoic acid
**23**	17.21	388.136	C_15_H_23_N_3_O_9_	2.8	C	D	(iso)valeryl CV	−0.4	
**24**	18.23	387.152	C_15_H_24_N_4_O_8_	3.1	C	E	(iso)valeryl VC	0.7	
**25**	19.48	387.150	C_15_H_24_N_4_O_8_	3.5	B	C	(iso)valeryl VC	−4.6	
derivatives with phenylpropanoids and guaiacylglycerol									
**26**	14.05	612.167	C_25_H_31_N_3_O_15_	8.8	A	D	CV coumaroyl Hex	−2.0	
**27**	14.59	611.181	C_25_H_32_N_4_O_14_	20.1	B	D	VC coumaroyl Hex	−4.7	
**28**	15.57	466.109	C_19_H_21_N_3_O_11_	4.1	A	D	CV caffeic acid	−2.4	
**29**	16.43	465.124	C_19_H_22_N_4_O_10_	4.4	B	E	VC caffeic acid	−4.1	
**30**	16.70	642.178	C_26_H_33_N_3_O_16_	8.7	A	D	CV feruloyl Hex	−1.2	
**31**	17.36	641.193	C_26_H_34_N_4_O_15_	9.9	A	D	VC feruloyl Hex	−3.5	
**32**	17.99	672.189	C_27_H_35_N_3_O_17_	3.9	C	D	CV sinapoyl Hex	−1.3	
**33**	18.42	671.203	C_27_H_36_N_4_O_16_	2.4	B	E	VC sinapoyl Hex	−4.2	
**34**	18.42	450.113	C_19_H_21_N_3_O_10_	8.0	C	E	CV coumaric acid	−4.8	
**35**	18.89	554.162	C_23_H_29_N_3_O_13_	3.9	A	D	CV + C_13_H_14_O_5_	−1.0	glyceryl ferulate
**36**	19.25	553.177	C_23_H_30_N_4_O_12_	3.3	A	D	VC + C_13_H_14_O_5_	−3.0	glyceryl ferulate
**37**	19.48	500.151	C_20_H_27_N_3_O_12_	6.8	E	D	CV + C_10_H_12_O_4_	−1.7	guaiacylglycerol
**38**	19.60	480.126	C_20_H_23_N_3_O_11_	2.9	A	E	CV ferulic acid	0.1	
**39**	19.99	479.141	C_20_H_24_N_4_O_10_	7.4	A	E	VC ferulic acid	−2.9	
**40**	20.03	499.166	C_20_H_28_N_4_O_11_	6.7	B	D	VC + C_10_H_12_O_4_	−4.2	guaiacylglycerol
**41**	20.14	510.135	C_21_H_25_N_3_O_12_	3.5	C	E	CV sinapinic acid	−3.9	
**42**	20.53	509.150	C_21_H_26_N_4_O_11_	5.4	B	E	VC sinapinic acid	−4.0	
**43**	20.97	676.197	C_30_H_35_N_3_O_15_	4.2	A	E	feruloyl CV + C_10_H_12_O_4_	−3.1	guaiacylglycerol
**44**	21.32	675.213	C_30_H_36_N_4_O_14_	7.8	A	E	feruloyl VC + C_10_H_12_O_4_	−3.3	guaiacylglycerol
**45**	22.57	1142.329	C_48_H_61_N_3_O_29_	9.1	A	E	CV diferuloyl triHex	−2.4	
**46**	22.60	980.278	C_42_H_51_N_3_O_24_	8.1	A	E	CV diferuloyl diHex	−1.3	
**47**	22.76	1141.345	C_48_H_62_N_4_O_28_	5.1	A	E	VC diferuloyl triHex	−2.3	
**48**	22.84	979.292	C_42_H_52_N_4_O_23_	12.9	A	E	VC diferuloyl diHex	−2.9	
**49**	22.96	818.224	C_36_H_41_N_3_O_19_	16.9	A	E	CV diferuloyl Hex	−2.8	
**50**	23.66	817.236	C_36_H_42_N_4_O_18_	2.8	B	E	VC diferuloyl Hex	−7.5	
**51**	25.04	1156.323	C_52_H_59_N_3_O_27_	3.3	A	D	CV triferuloyl diHex	−2.8	
**52**	25.23	1155.337	C_52_H_60_N_4_O_26_	3.7	A	E	VC triferuloyl diHex	−4.2	
Medium-chain acid (# C above 6) derivatives									
**53**	15.61	446.140	C_17_H_25_N_3_O_11_	3.6	A	D	CV + C_7_H_10_O_3_	−3.7	pimelic acid
**54**	16.62	445.156	C_17_H_26_N_4_O_10_	5.1	B	E	VC + C_7_H_10_O_3_	−4.7	pimelic acid
**55**	18.07	732.234	C_27_H_39_N_7_O_17_	10.5	B	E	CV + C_7_H_10_O_3_ VC	1.7	pimelic acid
**56**	19.32	789.247	C_30_H_42_N_6_O_19_	4.0	C	D	CV + C_10_H_14_O_4_ CV	4.3	hydroxy decenedioic acid
**57**	19.52	746.248	C_28_H_41_N_7_O_17_	2.0	A	D	VC + C_8_H_12_O_3_ CV	−1.5	suberic acid
**58**	19.83	788.261	C_30_H_43_N_7_O_18_	31.2	C	D	CV + C_10_H_14_O_4_ VC	2.9	hydroxy decenedioic acid
**59**	19.95	502.168	C_20_H_29_N_3_O_12_	6.6	C	D	CV + C_10_H_14_O_4_	0.3	hydroxy decenedioic acid
**60**	20.30	504.183	C_20_H_31_N_3_O_12_	16.1	C	D	CV + C_10_H_16_O_4_	−1.2	hydroxy sebacic acid
**61**	20.50	501.184	C_20_H_30_N_4_O_11_	9.4	C	D	VC + C_10_H_14_O_4_	−0.4	hydroxy decenedioic acid
**62**	20.61	572.211	C_24_H_35_N_3_O_13_	3.2	A	B	CV + C_14_H_20_O_5_	1.8	Dihydroxy-tetra-decadienedioic acid
**63**	20.77	503.199	C_20_H_32_N_4_O_11_	15.9	C	D	VC + C_10_H_16_O_4_	−0.7	hydroxy sebacic acid
**64**	20.85	472.156	C_19_H_27_N_3_O_11_	4.9	A	D	CV + C_9_H_12_O_3_	−2.1	nonenedioic acid
**65**	20.97	814.274	C_32_H_45_N_7_O_18_	24.4	A	E	VC + C_12_H_16_O_4_ CV	−1.5	hydroxy-dodecadienedioic acid
**66**	21.08	571.224	C_24_H_36_N_4_O_12_	2.8	A	B	VC + C_14_H_20_O_5_	−2.7	Dihydroxy-tetra-decadienedioic acid
**67**	21.24	474.173	C_19_H_29_N_3_O_11_	49.6	C	E	CV + C_9_H_14_O_3_	0.3	azelaic acid
**68**	21.28	813.288	C_32_H_46_N_8_O_17_	35.8	A	E	VC + C_12_H_16_O_4_ VC	−3.7	hydroxy-dodecadienedioic acid
**69**	21.43	471.171	C_19_H_28_N_4_O_10_	13.8	A	D	VC + C_9_H_12_O_3_	−5.0	nonenedioic acid
**70**	21.43	773.244	C_30_H_42_N_6_O_18_	6.5	A	D	CV + C_10_H_14_O_3_ CV	−5.3	decenedioic acid
**71**	21.47	528.180	C_22_H_31_N_3_O_12_	5.6	C	E	CV + C_12_H_16_O_4_	−6.8	hydroxy-dodecadienedioic acid
**72**	21.63	859.330	C_34_H_52_N_8_O_18_	10.7	B	E	VC + C_14_H_22_O_5_ VC	−3.1	dihydroxy-tetra-decenedioic acid
**73**	21.67	775.266	C_30_H_44_N_6_O_18_	4.1	A	B	CV C_10_H_16_O_3_ CV	2.7	sebacic acid
**74**	21.71	530.198	C_22_H_33_N_3_O_12_	2.5	C	E	CV + C_12_H_18_O_4_	−2.0	hydroxy-dodecenedioic acid
**75**	21.71	473.188	C_19_H_30_N_4_O_10_	2.5	C	E	VC + C_9_H_14_O_3_	−3.5	azelaic acid
**76**	21.94	772.265	C_30_H_43_N_7_O_17_	2.7	A	D	VC + C_10_H_14_O_3_ CV	0.4	decenedioic acid
**77**	22.02	527.198	C_22_H_32_N_4_O_11_	3.6	A	E	VC + C_12_H_16_O_4_	−3.3	hydroxy-dodecadienedioic acid
**78**	22.14	574.224	C_24_H_37_N_3_O_13_	2.9	A	E	CV + C_14_H_22_O_5_	−2.8	dihydroxy-tetra-decenedioic acid
**79**	22.17	774.281	C_30_H_45_N_7_O_17_	3.2	A	D	VC + C_10_H_16_O_3_ CV	1.0	sebacic acid
**80**	22.17	771.279	C_30_H_44_N_8_O_16_	3.0	A	E	VC + C_10_H_14_O_3_ VC	−2.1	decenedioic acid
**81**	22.17	529.213	C_22_H_34_N_4_O_11_	3.3	C	E	VC + C_12_H_18_O_4_	−3.8	hydroxy-dodecenedioic acid
**82**	22.37	573.239	C_24_H_38_N_4_O_12_	3.5	A	E	VC + C_14_H_22_O_5_	−3.7	dihydroxy-tetra-decenedioic acid
**83**	22.41	773.295	C_30_H_46_N_8_O_16_	4.1	A	D	VC + C_10_H_16_O_3_ VC	−1.2	sebacic acid
**84**	22.49	486.172	C_20_H_29_N_3_O_11_	3.0	A	B	CV + C_10_H_14_O_3_	−1.3	decenedioic acid
**85**	22.92	485.186	C_20_H_30_N_4_O_10_	2.1	A	E	VC + C_10_H_14_O_3_	−5.5	decenedioic acid
**86**	23.07	488.187	C_20_H_31_N_3_O_11_	5.3	A	D	CV + C_10_H_16_O_3_	−2.2	sebacic acid
**87**	23.27	485.187	C_20_H_30_N_4_O_10_	3.3	A	C	VC + C_10_H_14_O_3_	−3.2	decenedioic acid
**88**	23.58	487.203	C_20_H_32_N_4_O_10_	5.1	A	D	VC + C_10_H_16_O_3_	−3.2	sebacic acid

^a^: retention time in minutes; ^b^: based on the output of progenesis QI, the given *m*/*z* value corresponds to the experimental *m*/*z* of the [M − H]^−^ ion; ^c^: maximum fold change based on the average normalized intensity of the different varieties; ^d^: variety with the highest or lowest average MS signal intensity, details on the varieties A to E are given in Table 2; ^e^: mass error in ppm between the experimental *m*/*z* and the calculated *m*/*z* for the chemical formula in negative mode; ^f^: proposed name for the added group, determined based on mass/formula, the literature, and MS/MS fragments; VC: vicine; CV: convicine; Hex: hexose.

**Table 2:** The five faba bean varieties used in this study.

Code	Genotype	Registration GER	Color	Vicine	Type
A	Taifun	2011	Zt	HVC	Spring
B	Augusta	2018	Bb	HVC	Winter
C	Trumpet	2017	Bb	HVC	Spring
D	Allison	2019	Bb	LVC	Spring
E	Breeding line		Zt	LVC	Spring

## Data Availability

The data resulting in the here presented study can be found within the article or the Supplementary Materials. The identification database containing MS and MS/MS data as well as meta data concerning the identifications can be obtained on request to the corresponding author.

## Supplementary Materials

The following supporting information can be downloaded at https://www.mdpi.com/article/10.3390/molecules29051065/s1. Figure S1: Extracted fragment ion chromatograms of the fragment at 141.03 ± 0.015, covering the base peak fragment of vicine and convicine from the root samples and from the pool of extracts from the seedlings of 18 faba bean varieties. Table S1: Overview of 42 compounds showing the characteristic MS/MS signature of (con)vicine derivatives for which no identification was obtained. Table S2: Quantitative data on the 89 (con)vicine derivatives represented in Table 1, normalized quantitative data of the three replicates of the five varieties, and statistical data on the abundance of these compounds over the varieties; the *m*/*z* values of these fragments higher than 10% of the base peak were ordered from the most-intense to the least-intense are likewise provided.
