# Supplementary material for: Exploration of the Diversity of Vicine and Convicine Derivatives in Faba Bean (Vicia faba L.) Cultivars: Insights from LC-MS/MS Spectra
Source: Molecules. 2024 Feb 29;29(5):1065. doi: 10.3390/molecules29051065 (PMC10934171; doi:10.3390/molecules29051065)
Supplement: Supplementary file 1 [file molecules-29-01065-s001.zip › supplemental Figure S1.pdf]

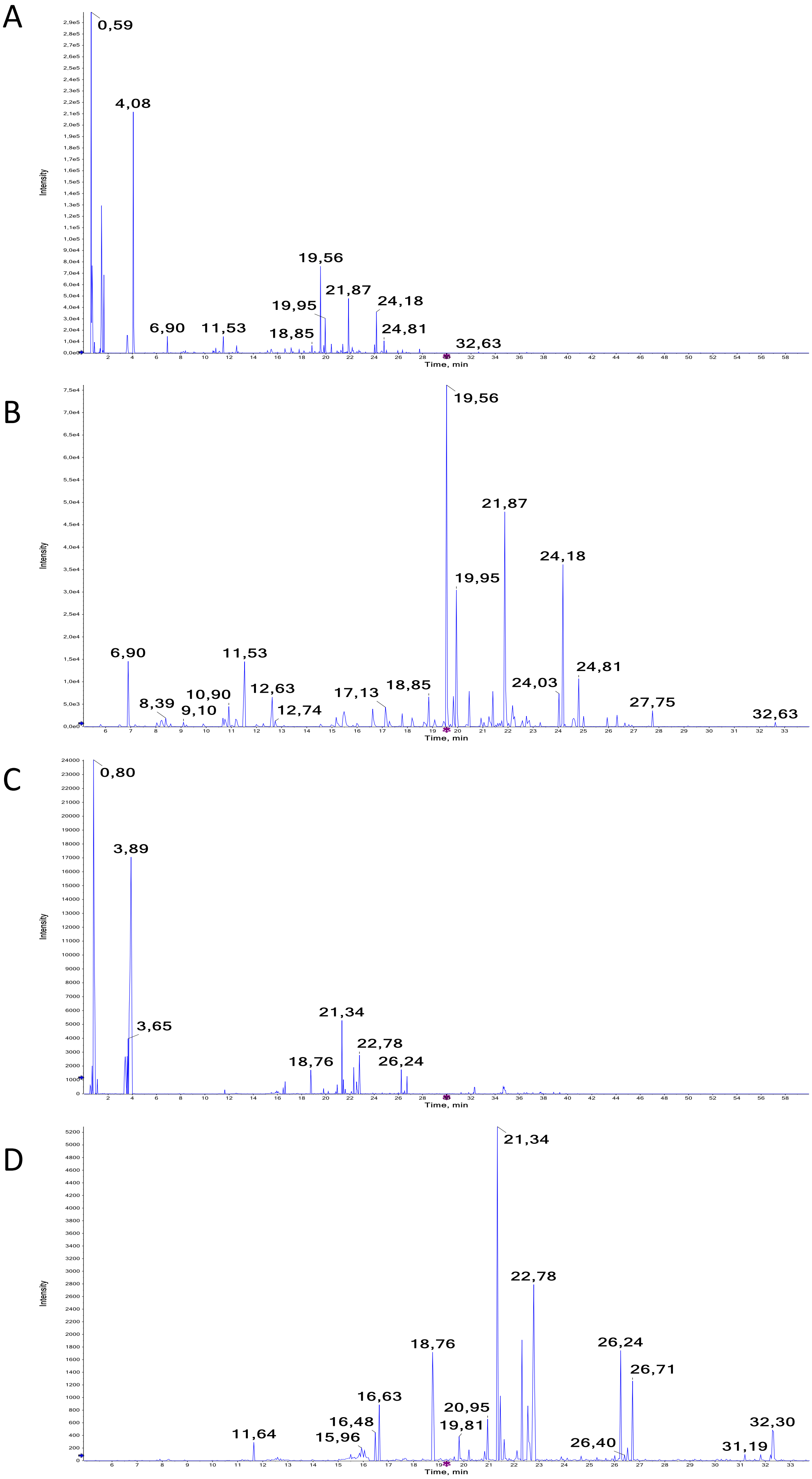

Supplemental Figure S1: Extracted fragment ion chromatogram of the fragment at 141.03+/-0.015, thus covering the base peak fragment of vicine and convicine. A &B: respectively full range and range from retention time 5 to 35 minutes of the roots sample from variety A. C&D: respectively full range and from retention time 5 to 35 minutes of the pool of extracts from 18 different faba bean varieties
